# Heat-related mortality and ambulance transport after a power outage in the Tokyo metropolitan area

**DOI:** 10.1097/EE9.0000000000000292

**Published:** 2024-02-19

**Authors:** Lisa Yamasaki, Takuma Kamada, Chris Fook Sheng Ng, Yuya Takane, Ko Nakajima, Kazuki Yamaguchi, Kazutaka Oka, Yasushi Honda, Yoonhee Kim, Masahiro Hashizume

**Affiliations:** a Department of Global Health Policy, Graduate School of Medicine, The University of Tokyo, Tokyo, Japan; b Center Hospital of the National Center for Global Health and Medicine, Tokyo, Japan; c Osaka School of International Public Policy, Osaka University, Osaka, Japan; d Environmental Management Research Institute, National Institute of Advanced Industrial Science and Technology (AIST), Tsukuba, Ibaraki, Japan; e TEPCO Research Institute, Tokyo Electric Power Company Holdings, Inc, Yokohama, Japan; f National Institute for Environmental Studies, Ibaraki, Japan; g Department of Global Environmental Health, Graduate School of Medicine, The University of Tokyo, Tokyo, Japan; h Institute of Tropical Medicine, Nagasaki University, Nagasaki, Japan

**Keywords:** Energy insecurity, Natural disasters, Power outage, Blackout, Hot temperature, Heat stress disorders, Ambulances, Mortality, Japan

## Abstract

**Background:**

Air conditioners can prevent heat-related illness and mortality, but the increased use of air conditioners may enhance susceptibility to heat-related illnesses during large-scale power failures. Here, we examined the risks of heat-related illness ambulance transport (HIAT) and mortality associated with typhoon-related electricity reduction (ER) in the summer months in the Tokyo metropolitan area.

**Methods:**

We conducted event study analyses to compare temperature–HIAT and mortality associations before and after the power outage (July to September 2019). To better understand the role of temperature during the power outage, we then examined whether the temperature–HIAT and mortality associations were modified by different power outage levels (0%, 10%, and 20% ER). We computed the ratios of relative risks to compare the risks associated with various ER values to the risks associated without ER.

**Results:**

We analyzed the data of 14,912 HIAT cases and 74,064 deaths. Overall, 93,200 power outage cases were observed when the typhoon hit. Event study results showed that the incidence rate ratio was 2.01 (95% confidence interval [CI] = 1.42, 2.84) with effects enduring up to 6 days, and 1.11 (95% CI = 1.02, 1.22) for mortality on the first 3 days after the typhoon hit. Comparing 20% to 0% ER, the ratios of relative risks of heat exposure were 2.32 (95% CI = 1.41, 3.82) for HIAT and 0.95 (95% CI = 0.75, 1.22) for mortality.

**Conclusions:**

A 20% ER was associated with a two-fold greater risk of HIAT because of summer heat during the power outage, but there was little evidence for the association with all-cause mortality.

**What this study adds**

To date, there has been limited quantitative evidence on the disaster-triggered health impact of power outages during hot weather. We used geographically fine-scale data on electricity use and explored the association of temperature modified by electricity use on morbidity and mortality in the largest metropolitan area in Japan. We found that that electricity reduction caused by a power outage led to a two-fold greater risk of heat-related morbidity during a power outage, while little evidence for association with all-cause mortality, highlighting the importance of additional precautions to avoid heat-related illness during power outages in hot weather.

## Introduction

As climate change intensifies, heat-related morbidity and mortality are becoming increasingly common problems worldwide.^1^ Exposure to extremely high ambient temperatures has been associated with a range of adverse health outcomes, accounting for nearly 1% of global deaths in the 20th century.^2^ During heatwaves, mortality increased by 3.7% compared with non-heatwave days in the United States,^3^ and Europe experienced over 60,000 deaths attributable to heat in the summer of 2022.^4^ Across the world, both extreme heat and heat waves are significantly linked to a rise in emergency ambulance dispatches.^5–7^ Specifically, the ambulance calls related to heat-related illness including heat stroke, the forecast for 2100 suggests a six-fold increase compared with 2000, with a significant rise expected among the elderly population in Japan.^8^ Another investigation suggested that the incidence of heatstroke for people aged 65 years is projected to be 3.26 and 1.69 at the end of the 21st century with and without human adaptation respectively, in comparison to the period 1981–2000.^9^

Air conditioning use is an effective adaptation to prevent heat-related illness and mortality.^10^ With income growth around the world, the availability and global use of air conditioning is expected to increase considerably over the coming years in response to rising global temperatures.^11^ Assuming a more extreme climate in the future, preventing further heat-related risks may require upscaling of interventions that involve air conditioning.

While the proliferation of air conditioning systems is desirable, increasing reliance on the power supply can increase vulnerability to heat-related illnesses in the event of large-scale power failures. Power supply interruptions have been associated with increases in all-cause mortality,^12^ hospital admissions for respiratory disease,^13^ emergency department visits,^14,15^ and overwhelmed emergency medical services.^16,17^

The double hazards of global warming and typhoons (also known as tropical cyclones and hurricanes) have been highlighted recently in response to the substantial increase in the frequency of weather-related disasters.^18^ Because most reported power outage events are caused by severe weather,^19^ the importance of disaster preparedness against compound climate and infrastructure failure events has been emphasized.^20^ In Japan, half of annual large-scale power outages (11 of 22 cases) were caused by natural disasters.^21^ In the era of climate change, an assessment of how impaired access to electricity on hot days can cause health problems is critically needed to inform the development of public health interventions in response to disasters.

To our knowledge, there has been limited research concerning the health impact of electricity reduction (ER) caused by power outages during hot seasons.^22,23^ For instance, Dominianni et al focused on power outages resulting from both disasters and manmade demand for electric power and examined the relationship between power outages and mortality using time-series analyses.^17^ Another study estimated that an energy-saving strategy resulting from the Great East Japan earthquake resulted in more than 7,000 premature deaths per year in Japan.^23^ The present study builds on this literature and aims to make three contributions. First, it focuses on a natural disaster-induced power outage. Our empirical setting is a disruption of electricity caused by a typhoon that hit 46 districts in the Tokyo metropolitan area during the summer of 2019, resulting in 93,200 power outage cases, thereby generating a natural experimental variation. Importantly, the daily average temperature was very similar between power outage-affected districts and nonaffected districts before and after the typhoon. Thus, conditional on baseline district characteristics, the occurrence of power outage was plausibly exogenous to the outcomes of interest. Second, exploiting this natural experimental variation, we applied an event study design, which is based on a difference-in-differences (DiD) framework. We estimated the dynamic effects of a power outage on the outcomes of interest in the days leading to and following a power outage. Additionally, electricity consumption data from the electricity company was used to further consider the impact of heat at varying degrees of ER, instead of a binary indicator as in previous studies.^12–17,24^ To understand how power outages and summer heat combine to affect human health, we focused on two commonly studied health outcomes: —heat-related illness ambulance transport (HIAT) and mortality.

## Methods

### Study area

**Figure 1.**
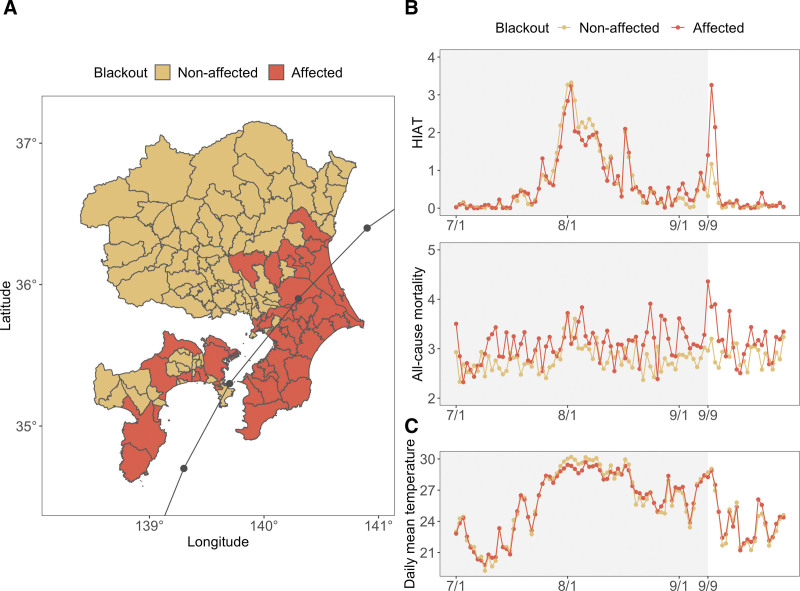
Typhoon-triggered power outages and trends of health outcomes and temperature from July 2019 to September 2019. A, Typhoon trajectory and power outage-affected areas. The typhoon trajectory is represented by a line with dots every 3 hours from 3 pm on September 8 to 12 am on September 9. B, Trends in heat-related illness ambulance transport (HIAT) and all-cause mortality. The daily incidences of HIAT and mortality in each district were averaged independently for affected and nonaffected areas. C, The average daily mean temperatures in affected areas and nonaffected areas.

The study area encompasses seven prefectures in the Tokyo metropolitan areas (Ibaraki, Tochigi, Gunma, Saitama, Chiba, Kanagawa, and Shizuoka). Tokyo was excluded because the HIAT data were unavailable. Data were available at the fire department district level, each containing one to several cities served by a fire department (Figure 1A). In total, 136 districts across seven prefectures were analyzed. The typhoon hit on 9 September 2019, and of 136 districts, 46 were affected by a power outage.

### Data

We collected data on the daily counts of HIAT spanning 3 months from July to September 2019 from the disaster prevention departments in each prefecture, which managed emergency transportation. The diagnoses of patients with various disorders, including heat-related illnesses were determined through expert judgment during initial assessments by the physicians. The disaster prevention department is in charge of emergency ambulance transport. Heat-related illness was coded according to the International Classification of Diseases, 10th Revision (ICD-10), including categories such as “heatstroke and sunstroke” (T67.0), “heat syncope” (T67.1), “heat cramp” (T67.2), “anhidrotic heat exhaustion” (T67.3), “unspecified heat fatigue” (T67.5), “heat fatigue, transient” (T67.6), “heat edema” (T67.7), and “other effects of heat and light” (T67.8). Daily mortality data for the same period and locations were obtained from the Ministry of Health, Labour and Welfare of Japan. The study period was selected based on the three highest monthly average temperatures during summer. Daily data on electricity consumption was prepared by our previous works^25,26^ and the number of power outage contract cases was provided by the Tokyo Electric Power Company (TEPCO) Power Grid,^27^ which supplies electricity to the study area. Electricity consumption data were used to calculate ER caused by the power outages at the fire department district level (Figure S1; http://links.lww.com/EE/A261). To supplement the information on ER, each power outage case at the household level was used as a binary indicator of the existence of a power outage. ER was defined as the magnitude of a power outage when power outage cases (each case with more than 12 hours of a power outage) were >0; we assumed that a power outage event ended when the number of power outage cases reached zero. We collected daily weather data (e.g., daily mean temperature and relative humidity) from the Japan Meteorological Agency. The daily mean temperature in 2019 is comparable across the recent of 2015–2019 (Figure S2; http://links.lww.com/EE/A261).

### Statistical analysis

We conducted two sets of empirical analyses. First, we used an event study design to examine the effects of the power outage event on health-related outcomes in the days leading to and following the power outage. Second, to better understand the potential modifying effect of power outage on the risk of heat exposure, we quantified the risk of temperature exposure under different power outage levels determined using a continuous variable for district-level ER, which was regarded as a potential effect modifier. We incorporated possible delayed effects of temperature using a distributed lag nonlinear model (DLNM).

### Event study analysis

We used an event study design to explore the time-varying effects of a typhoon-triggered power outage on HIAT and all-cause mortality. The event study design, which is built on a DiD framework, estimates dynamic treatment effects over time with leading and lagged treatment indicators. First, with leading treatment indicators, we indirectly test the parallel trend assumption, a crucial identifying assumption in the DiD design. It states that in the absence of a power outage, outcome trends would change at similar rates in the affected and nonaffected districts. If the parallel trends assumption holds, the estimated coefficients of event indicators in the pre-power outage period (i.e., leading treatment indicators) should be statistically indistinguishable from zero. Second, we include lagged treatment indicators, which allow us to explore whether the effects of the power outage were immediate and attenuated, persisted, or escalated over time. Here, we estimated the following event study specification using a Poisson-pseudo maximum likelihood estimator.^28–30^ This estimator has two advantages. First, it is consistent under mild distributional assumptions, so long as the conditional expectation is correctly specified. Second, it does not suffer from the incidental parameters problem, and we can thus control for district-level fixed effects.


$$\begin{array}{l} log\;E\left(Y_{i,j,t}|Z_{i,j}{,}_{t}\right)=\overset{6}{\underset{\tau =-20,\tau \neq -1}{\sum }}\alpha_{\tau }\times (1_{i}^{power}\times 1_{t}^{\tau })+\ln Pop_{i}+Covariate_{i,t}+\mu_{i}+\gamma_{j,t}+\epsilon_{i,j,t} \end{array}$$ (1)

where $Y_{i,t}$ is the outcome of interest: the daily number of HIAT or all-cause mortality in district i, prefecture j and day t. $Z_{i,j}{,}_{t}$ includes all variables on the right side of the equation. We normalized the timing of observation, such that time *t* is measured with respect to the day of the power outage. $1_{t}^{\tau }$ is a pre- and post-time indicator that equals to 1 in a relevant τ lead or τ lag period. An interval of three days is set (days 0–2, 3–5, 6–8, …) to observe time-varying effects during the post-power outage period (September 9 to 30). The set of time indicators is interacted with $1_{i}^{power}$, an indicator that equals 1 for power outage-affected districts and is otherwise 0. $\alpha_{\tau }$ represents the coefficients of interest, which capture the dynamic effects of the power outage in the days (each 3-day interval) leading to and following the power outage. $\mu_{i}$ represents individual district fixed effects, which control for time-invariant factors, such as demographic and social characteristics and infrastructures, at the district level during the study period. $\gamma_{j,t}$ denotes prefecture-day fixed effects, controlling for any arbitrary time-varying shocks specific to prefectures. This allows us to compare affected districted and nonaffected districted within the prefecture. $pop_{i}$ is a population in the district i. Humidity and DLNM for temperature are specified as $Covariate_{i,t}$. DLNM was employed to investigate the bidimensional exposure-lag-response associations between temperature and health outcomes. A natural cubic spline function was applied to the temperature variable with 1 internal knot. For the lag dimension, we specified a lag period of 1 for HIAT^31^ and mortality,^32^ to capture possible delayed effects of heat exposure. The average of lag 0 to 1 was used. $\epsilon_{i,t}$ is the error term. Standard errors are clustered at the district level to account for serial correlation within the district.

### Effect modification analysis

To investigate the modifying effect of power outages on heat-health associations during typhoons, we employed an interaction model in which we included the interaction terms of temperature and ER. ER is the percentage difference between predicted electricity consumption and observed electricity consumption.^33^ The model for predicting electricity consumption for the post-typhoon period included variables for the daily mean temperature, humidity, day of the week, day of the season, and calendar year (Figure S3; http://links.lww.com/EE/A261). Before the typhoon, ER was restricted to zero.^33^ A linear interaction term was generated by multiplying the crossbasis of temperature with ER.^33–35^ The cumulative relative risks (RRs) of health outcomes were estimated by centering ER at 10% and 20%. We specified our model as follows:


$$\begin{array}{l} log\;E\left(Y_{i,t}|Z_{i,t}\right)=\alpha_{1}\times ER_{i,t}+\alpha_{2}\times {Temp}_{i,t}+\alpha_{3}\times ER\cdot Temp_{i,t}\;\\ \;+\alpha_{4}ln\;Pop_{i}\;+\;\alpha_{5}\;\times \;Humidity_{i,t}+\alpha_{6}\;\times \;DOW_{t}+\alpha_{7}\times Holiday_{t}\;\\ \;+\alpha_{8}\times ns\left(DOY,3\right)+\mu_{i}+\epsilon_{i,t} \end{array}$$ (2)

In this equation, $Y_{i,t}$ represents the outcomes of interest in district i and day t, $Z_{i,t}$ includes all variables on the right side of the equation. $ER_{i,t}$ is ER representing power outage magnitude with district-level ER. For temperature ($Temp_{i,t}$), the same DLNM was employed as described above. $Pop_{i}$. $Humidity_{i,t}$, $DOW_{t}$_,_ and $Holiday_{t}$ represent a population, relative humidity, day of week, and holiday, respectively, and were adjusted for as confounders. We considered possible time-varying effects on health outcomes instead of using a time-fixed effect to capture the impact of temperature. A natural cubic spline with three equally spaced knots for the day of the year (DOY), consecutive numbers representing each day from July to September in 2019) was used to model seasonality following the standard model specification in the epidemiological literature.^36,37^ Using this approach, we estimated the RRs for heat (95th vs. 50th percentile of daily average temperature) at different ER values (0%, 10%, and 20%). The ratios of RRs (RRRs) were computed to compare the risks associated with various ER values to the risks associated with no ER in the absence of a power outage.^38^ We also performed the subgroup analysis by transport location (i.e., home, work and school, and public space), by sex and by age group (i.e., 0–64 years old, and 65 years and older).

For the sensitivity analyses, we repeated the analyses for the baseline by increasing the degrees of freedom for temperature to 3 and 4 (Figure S4; http://links.lww.com/EE/A261). All analyses were performed using R (version 4.1.3). The “*glm*” package was used for regression analysis. Cluster-robust standard error was calculated using the “*lmtest*” package. The DLNM was implemented using the “*dlnm*” package.

## Results

**Figure 2.**
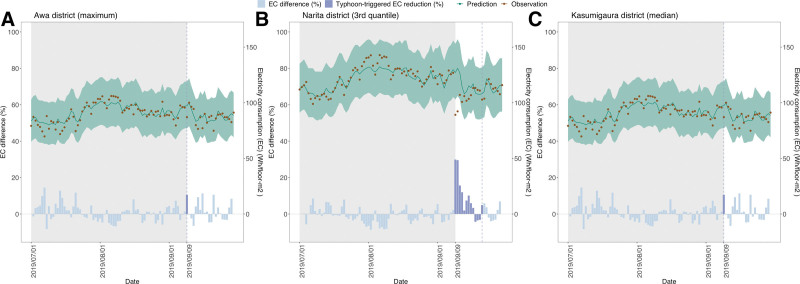
Estimated reduction of electricity use at the district level. A, Awa district exhibited the highest electricity reduction among all fire departments on the day that the typhoon hit. B, Narita district exhibited electricity reduction in the 3rd quantile on the day that the typhoon hit. C, Kasumigaura district exhibited the median electricity reduction. Green lines show predicted values, and the shaded area indicates prediction intervals of predicted values. Daily observed electricity use is shown as solid dots in brown. The blue bars at the bottom represent percent reductions between observed electricity use (brown dot) and predicted electricity use (green line). The period with typhoon-triggered electricity reduction, defined based on preliminary reports from Tokyo Electric Power Company, is shown as a blue dotted vertical line that represents the day when the number of power outages is zero.

We analyzed the data from 14,912 HIAT cases and 74,064 deaths from July 2019 to September 2019. A total of 93,200 power outage cases at the household level were observed on September 9 when a typhoon hit Japan in 46 districts. On the day that the typhoon approached, average daily mean temperatures of 28.3 °C and 28.6 °C were observed in the power outage-affected and nonaffected districts, respectively. The ERs in three districts with different levels of power outage (the 100th, 75th, and 50th percentiles of ER), along with the predicted and observed values of electricity use, are shown in Figure 2. The most affected area was Awa district, which had 88% ER on the day that the typhoon hit. During both the pre- and post-power outage periods, individuals aged 65 and older show a higher occurrence of both HIAT and mortality, with a higher proportion of males than females. The majority of HIAT incidents occur at home, and these findings are outlined in Table S1; http://links.lww.com/EE/A261.

**Figure 3.**
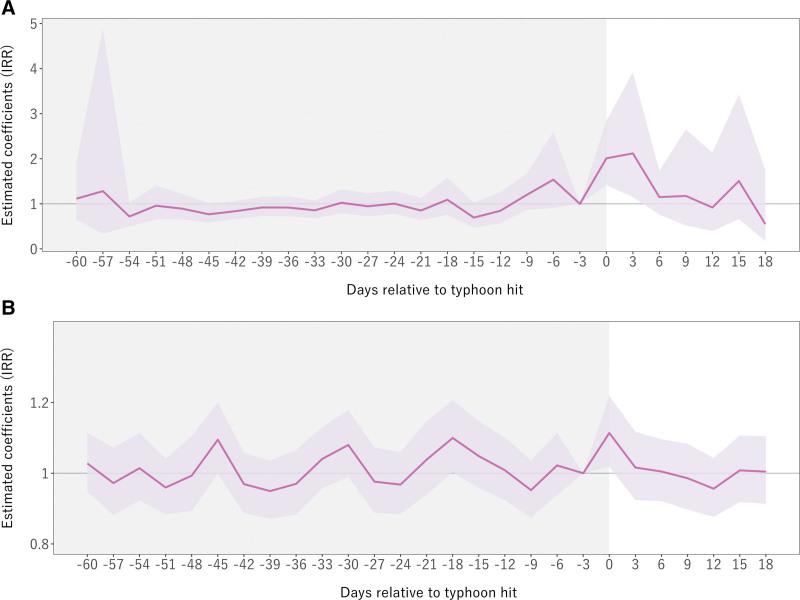
Event study analysis of heat-related illness ambulance transport (HIAT) and all-cause mortality in the pre- and post-power outage period. The purple line shows the incidence rate ratio of interaction terms (binary indicators of each period and blackout-affected area) in the event study analysis, and 95% confidence interval is shaded. The period before the blackout intervention is shaded in gray. Each number in *x* axis includes 3 days. A, Heat-related illness ambulance transport (HIAT) and (B) All-cause mortality.

Figure 3 graphically provides exponentiated estimated coefficients and 95% confidence intervals (CIs), and Table 1 provides their estimates in value. Figure 3A represents the effects on HIAT, and Figure 3B represents the effects on all-cause mortality rates.

**Table 1.** Incidence rate ratio (IRR) in the post-power outage period compared with that in the reference period (one period before the power outage) for heat-related illness ambulance transport (HIAT) and all-cause mortality

Event study results						
	HIAT			All-cause mortality		
	Control	Power outage	IRR (95% CI)	Control	Power outage	IRR (95% CI)
Pre-power outage (reference)						
Day −3 to −1	0.58	0.64	–	2.82	3.12	–
Post-power outage						
Day 0–2	0.72	2.27	2.01 (1.42, 2.84)^b^	2.96	4.04	1.11 (1.02, 1.22)^a^
Day 3–5	0.06	0.18	2.12 (1.15, 3.92)^a^	2.84	3.06	1.02 (0.92, 1.12)
Day 6–8	0.13	0.15	1.15 (0.76, 1.73)	3.02	3.12	1.00 (0.92, 1.10)
Day 9–11	0.03	0.04	1.17 (0.52, 2.65)	2.65	2.82	0.99 (0.90, 1.08)
Day 12–14	0.03	0.05	0.92 (0.39, 2.13)	3.02	3.23	0.96 (0.88, 1.04)
Day 15–17	0.07	0.19	1.51 (0.66, 3.43)	2.75	3.04	1.01 (0.92, 1.11)
Day 18–20	0.08	0.07	0.55 (0.17, 1.75)	2.88	3.12	1.00 (0.91, 1.10)

Results for “control” and “power outage” show the average incidences of HIAT and all-cause mortality per 100,000 in each 3-day interval in the control (nonaffected) group and power outage (affected) group.

a *P* < 0.05.

b *P* < 0.01.

First, the event study result in Figure 3A corroborates the descriptive pattern shown in Figure 1B (the top panel), which presents HIAT in the affected and nonaffected districts over time. Figure 3A indicates that trends in HIAT were similar between the affected districts and nonaffected districts before the power outage. In the days following the power outage, HIAT increased in the affected districts relative to the nonaffected districts, but the increased trends reverted 6 days after the power outage. Specifically, HIAT cases increased by 101% (95% CI = 42%, 184%) during the first 3-day period (day 0–2) of a power outage, as denoted by incidence rate ratio (IRR)^39^ of 2.01 (95% CI = 1.42, 2.84) (Table 1). During the next 3-day period (days 3–5), HIAT increased by 112% (95% CI = 15%, 292%). However, there were negligible differences in HIAT between the affected and nonaffected districts after 6 days and onward.

Second, the event study result in Figure 3B generally corroborates the descriptive pattern shown in Figure 1B (the middle panel). Figure 1B indicates a spike in all-cause mortality in the affected districts relative to the nonaffected districts on the day of the power outage; however, note that the affected districts exhibited a higher number of all-cause mortality than the nonaffected districts before the power outage. This implies that, despite very similar temperatures between affected districts and nonaffected districts in the pre-power outage period, high all-cause mortality in the affected districts in the post-power outage period might have been driven by preexisting upward trends in the pre-power outage period. Consistent with this, Figure 3B, where we used the 1–3 days before the power outage as a reference period, shows a spike (albeit small) in all-cause mortality only in the first three days after the power outage. In Table 1, the IRR for all-cause mortality was 1.11 (95% CI = 1.02, 1.22) during the same period. Subgroup analysis of event study for HAIT and all-cause mortality by age are shown in (Figure S5; http://links.lww.com/EE/A261 and Figure S6; http://links.lww.com/EE/A261).

**Figure 4.**
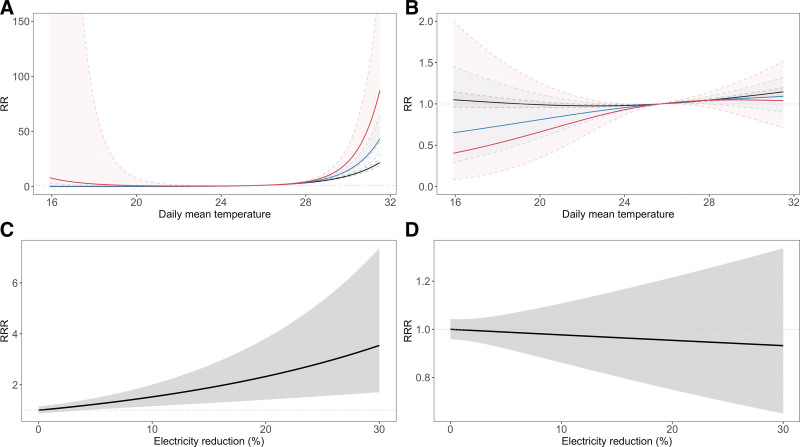
Relative risk (RR) of health outcomes for temperature and the ratio of RR (RRR) with different electricity reduction percentages. Cumulative exposure-response curves exhibit the effects of temperature and outcomes: (A) Heat-related illness ambulance transport (HIAT) and (B) All-cause mortality. The black line shows 0% electricity reduction (ER), the blue line shows power outage at 10% ER, while the red line shows that at 20% ER. The dotted lines represent 95% confidence intervals. RRR of health outcomes for exposure to heat (95th percentile vs. 50th percentile of daily mean temperature, 30 °C vs. 25.7 °C) for different electricity reduction percentages in comparison to the reference of no electricity reduction (normal days without power outage) for (C) HIAT and (D) All-cause mortality. Shaded areas represent 95% confidence intervals.

We next turned our attention to effect modification analyses to better understand the role of heat during the power outage. Note that unlike previous event study analyses, where we used a binary indicator for the power outage, we focused on the different levels of the power outage. Exposure-response curves are presented for HIAT and all-cause mortality to examine effect modification at three different power outage levels (0%, 10%, and 20% ER) (Figure 4A, B). Without power outage, the RRs for heat (95th vs. 50th percentile of temperature) were 9.79 (95% CI = 8.87, 10.80) and 1.10 (95% CI = 1.07, 1.13) for HIAT and all-cause mortality, respectively. When ER was 20%, the RRs were 22.60 (95% CI = 13.89, 36.79) for HIAT and 1.04 (95% CI = 0.82, 1.33) for all-cause mortality. The RRRs of heat exposure comparing 10% to 0% ER were 1.52 (95% CI = 1.16, 2.01) for HIAT and 0.98 (95% CI = 0.86, 1.11) for all-cause mortality (Figure 4). When ER increased to 20%, the RRRs were 2.32 (95% CI = 1.41, 3.82) for HIAT and 0.95 (95% CI = 0.75, 1.21) for all-cause mortality (Table 2). Figure 4 shows approximately linear associations of RRR for HIAT and all-cause mortality.

**Table 2.** The ratio of relative risk (RRR) by subgroups for HIAT and mortality associated with exposure to heat (95th percentile vs. 50th percentile of daily mean temperature, 30 °C vs. 5.7 °C) at 10% and 20% electricity reduction in comparison to the reference of no electricity reduction (normal days without power outage)

Ratio of relative risk				
Category		0% (reference)	10% (95% CI)	20% (95% CI)
HIAT				
All		1.00	1.52 (1.16, 2.01)^a^	2.32 (1.41, 3.82)^a^
Place	Home	1.00	1.36 (0.97, 1.91)	1.85 (1.03, 3.33)^a^
	Work or school	1.00	1.13 (0.58, 2.21)	1.27 (0.37, 4.44)
	Public places	1.00	1.88 (1.03, 3.43)^a^	3.54 (1.16, 10.78)^a^
Sex	Male	1.00	1.22 (0.88, 1.69)	1.49 (0.83, 2.69)
	Female	1.00	2.17 (1.41, 3.36)^a^	4.72 (2.15, 10.37)^a^
Age	0–64	1.00	1.69 (1.08, 2.65)^a^	2.86 (1.24, 6.62)^a^
	65+	1.00	1.49 (1.07, 2.07)^a^	2.21 (1.24, 3.96)^a^
Mortality				
All		1.00	0.98 (0.86, 1.11)	0.95 (0.75, 1.22)
Cause	Cardiovascular	1.00	1.07 (0.84, 1.36)	1.14 (0.72, 1.8)
	Respiratory	1.00	0.89 (0.65, 1.21)	0.78 (0.43, 1.42)
	Other	1.00	0.96 (0.81, 1.14)	0.92 (0.67, 1.28)
Sex	Male	1.00	0.99 (0.83, 1.17)	0.97 (0.7, 1.35)
	Female	1.00	0.97 (0.81, 1.16)	0.93 (0.66, 1.33)
Age	0–64	1.00	0.92 (0.6, 1.39)	0.84 (0.37, 1.89)
	65+	1.00	0.98 (0.86, 1.12)	0.97 (0.75, 1.25)

a *P* < 0.05.

**Figure 5.**
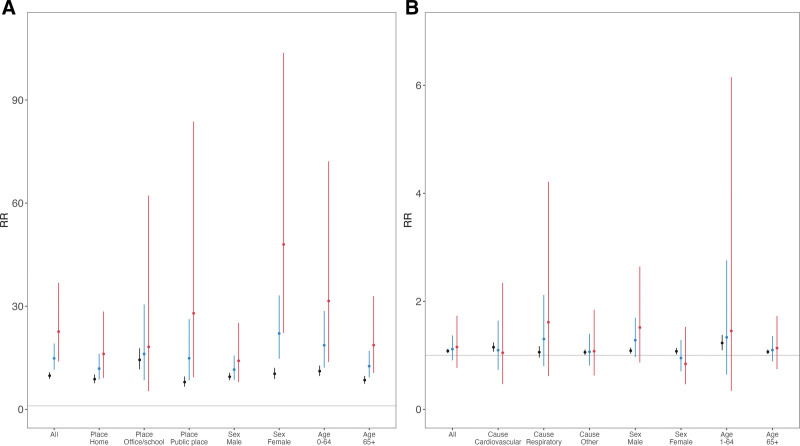
The relative risks (RR) for heat (95th percentile vs. 50th percentile of daily mean temperature, 30 °C vs. 25.7 °C) for different subgroups of health outcomes. A, Presents HIAT and (B) Presents all-cause mortality. The black line shows 0% electricity reduction (ER), the blue line shows power outage at 10% ER, and the red line shows that at 20% ER.

Lastly, the RRs for subgroups are presented in Figure 5. For HIAT cases transported from home and public places, the RRR comparing the risk of heat exposure at 20% ER to the risk on normal days without any ER was 1.85 (95% CI = 1.03, 3.33) and 3.54 (95% CI = 1.16, 10.78), respectively (Table 2). The RRRs for HIAT cases among women also exceeded 1 for both 10% and 20% ER at 2.17 (95% CI = 1.41, 3.36) and 4.72 (95% CI = 2.15, 10.37). The same pattern was observed in the individuals aged 0–64 years old and those 65 years and older for the HIAT outcome. The exposure-response curves are shown in Figure S7; http://links.lww.com/EE/A261 and Figure S8; http://links.lww.com/EE/A261 respectively, for HIAT and mortality. For both health outcomes, the exposure-response curves did not change substantially when the degrees of freedom for the temperature were increased (Figure S4; http://links.lww.com/EE/A261).

## Discussion

This study examined the health impacts of a natural disaster-triggered power outage and explored the role of power outage on heat-related health risks using district-level electricity consumption data in the Tokyo metropolitan area.

Extreme heat events are the main cause of weather-related mortality and ambulance transport worldwide including the study area as Tokyo is one of the heat-affected areas in the world of urban heat islands with high population concentration.^40^ Substantial power outages, ranging from 0.9 to 8.5 billion customers of lost supply, have already resulted from previous tropical cyclones.^41–43^ Tropical cyclones can extend their impact beyond areas lacking sufficient power supply to include well-powered temperate zones, affecting people through increased heat in a changing climate.^44^ Although heat-related mortality was not previously reported during mega-blackouts caused by tropical cyclones, there is a rapid and nonlinear rise in the number of compound tropical cyclones and heat events, with the escalation of global warming.^45^

Our event study analyses showed that the event of power outage doubled HIAT during the first 3 days in power outage-affected areas; this increase lasted until 6 days after the typhoon hit. For all-cause mortality, there was a much smaller effect–it increased 11% only in the first 3 days. The results of effect modification analyses showed that HIAT was associated with high ER; however, all-cause mortality was not associated with heat exposure at any level of ER. Gaps in the results of the association of heat with morbidity and mortality have been previously reported: Previous investigations generally did not find an impact of heat spells on morbidity (hospital admissions) despite the impact observed on mortality.^46–48^ Another study showed a diverse result, in that heat-related morbidity was reported.^49^ In our study, we use heat-related ambulance transport as a marker for morbidity, which might make a clear association for transportation rather than mortality.

In the analysis according to the location of transport, HIAT cases transported from home and public places were associated with a higher risk for heat at 20% ER, likely because during typhoons when power outages occur, people were advised to stay at homes or in public institutions designated as evacuation centers (versus office or school) which are at risk of power failure unless they evacuate to somewhere safe. Many might not commute during a typhoon and might stay put over the next several days depending on severity. HIAT cases among women also revealed substantial vulnerability to the power outage (20% ER), compared with the absence of the power outage; this led to a 4.7-fold higher risk of heat exposure. Although the absolute number of HIAT cases was higher for men than women partially related to demographic and professional factors,^50,51^ some studies suggest that women are more sensitive to high temperatures for cardiovascular diseases.^52,53^ Our analysis indicated that when a power outage occurs, a dramatic increase in HIAT among women may occur. A possible reason might be a higher proportion of older females than men, but more data with detailed age information for each sex will be necessary to examine this thoroughly. Despite the biological vulnerability of older adults to heat,^50^ we observed considerable RRRs to heat exposure upon ER in both age groups. The increased risk particularly among individuals aged 0–64 years old may be related to inadequate heat preparedness, more physical work, or increased exposure to heat.

In the official Disaster Prevention and Crisis Management report for Chiba prefecture,^54^ where the most severe power outages occurred, a total of nine deaths were registered as disaster-related deaths based on an official investigation by the local disaster management department for monetary support. Among these, seven deaths were officially reported as power outage-related, one resulted from the inaccessibility of a home oxygen inhaler powered by electricity, while the remaining case was attributed to heat-related illnesses and other factors stemming from power outages. This report shows that the number of deaths directly caused by the typhoon, such as trauma and drowning, was limited, as most cases were linked to the inaccessibility of electricity.

Our findings are consistent with these reports as the power outages were found little effect on mortality, although no modification of the association between mortality and temperature by the different levels of the power outage was observed. A previous study also showed that sudden power outages impeded the use of medical devices powered by electricity.^15^ Despite this similarity, other factors that determine the health impact of a power outage include the timing and cause of the power outage. For instance, a previous large power outage event,—the 2003 power outage in the northern United States had distinct characteristics compared with the power outage analyzed in the present study. First, the northern United States power outage happened in the evening when the temperature was generally moderate. Therefore, in the previous study evening temperatures were not regarded as a primary mechanism that affected mortality.^12^ In terms of temperature, power outage events in winter also cause a different set of problems such as an increase in emergency department visits.^14^ There might be a difference in physiological adaptation between the beginning and end of seasons (e.g., a higher risk of mortality was observed in early summer compared with late summer when exposed to the same extent of heat^34^). The northern United States power outage happened at a time when people were active in the community; for example, 800 elevator rescues were performed,^55^ and 350,000 individuals were on subway trains when the power outage began.^56^ This timing carries the additional risk of accidental mortality.^12^ Second, the power outage in the present study was caused by a natural disaster, whereas the northern United States power outage was caused by a technical failure. Factors that may contribute to health problems may differ between these two origins; for example, more severe traffic issues (e.g., flooded roads with fallen trees) could impede the transport of patients with acute symptoms during naturaldisaster-related power outages.

There were some limitations in this study. First, this study comprised a district-level analysis, and it did not investigate individual-level associations. It is therefore unknown whether individuals were exposed to heat or ER. Second, because electricity consumption data includes the use of air conditioners, lights/appliances, and cooking equipment, the results should be interpreted as the overall impact of ER in the area. Third, other influencing factors such as socioeconomic factors and the prevalence of household air conditioning may exist. Although the usage of air conditioning during the event is unknown, the prevalence of household air conditioning in the study area is relatively high, surpassing 92.6% in 2016.^57^ Fourth, diagnostic accuracy may have been biased because some ambulance transport cases did not involve diagnosis by physicians; on hot days in-depth examinations were unnecessary to establish a diagnosis of heat-related illness. Lastly, in event study analyses, we did not address the intensity or duration of a power outage.

Overall, after the power outage, increased HIAT cases were observed as IRRs greater than 2 and lasting 6 days while a slight increase in mortality was observed in the first 3 days. The reduction of electricity use by 20% was associated with a two-fold higher risk of HIAT because of summer heat in the metropolitan areas of Japan, but there was little evidence of an increased risk of mortality. Following the current concern about global warming and extreme climate events, public health authorities at the national, subnational, and local levels should be encouraged to promote public preparedness and mitigate disaster-related hazards of heat during power outages.

## Acknowledgments

We thank the fire departments of Chiba, Kanagawa, Tochigi, Ibaraki, Shizuoka, Gunma, Saitama prefectures for providing data on heat-related ambulance transport; Hiroaki Murayama for his input on methodology; and Shigeru Nagata for providing us data on power outage cases.

## Conflicts of interest statement

The authors declare that they have no conflicts of interest with regard to the content of this report.

## Funding

M.H. is supported by the Japan Science and Technology Agency (JST) as part of SICORP, Grant Number JPMJSC20E4 and the Environment Research and Technology Development Fund (JPMEERF20231007) of the Environmental Restoration and Conservation Agency provided by Ministry of the Environment of Japan. Y.T. and K.N. are supported by the Environmental Research and Technology Development Fund (JPMEERF20191009) of the Environmental Restoration and Conservation Agency of Japan and JSPS KAKENHI Grand Number JP20KK0096. K.O. is supported by the Climate Change Adaptation Research Program of the National Institute for Environmental Studies.

## Supplementary Material



## References

[1] Gasparrini A Guo Y Sera F. Projections of temperature-related excess mortality under climate change scenarios. Lancet Planet Health. 2017;1:e360–e367. 29276803 10.1016/S2542-5196(17)30156-0 PMC5729020

[2] Burkart KG Brauer M Aravkin AY. Estimating the cause-specific relative risks of non-optimal temperature on daily mortality: a two-part modelling approach applied to the global burden of disease study. Lancet. 2021;398:685–697. 34419204 10.1016/S0140-6736(21)01700-1 PMC8387975

[3] Khatana SAM Werner RM Groeneveld PW. Association of extreme heat with all-cause mortality in the contiguous US, 2008-2017. JAMA Netw Open. 2022;5:e2212957–e2212957. 35587347 10.1001/jamanetworkopen.2022.12957 PMC9121188

[4] Ballester J Quijal-Zamorano M Méndez Turrubiates RF. Heat-related mortality in Europe during the summer of 2022. Nat Med. 2023;29:1857–1866. 37429922 10.1038/s41591-023-02419-z PMC10353926

[5] Onozuka D Hagihara A. Spatial and temporal variation in emergency transport during periods of extreme heat in Japan: a nationwide study. Sci Total Environ. 2016;544:220–229. 26657368 10.1016/j.scitotenv.2015.11.098

[6] Cheng J Xu Z Zhao D. The burden of extreme heat and heatwave on emergency ambulance dispatches: a time-series study in Huainan, China. Sci Total Environ. 2016;571:27–33. 27454572 10.1016/j.scitotenv.2016.07.103

[7] Schaffer A Muscatello D Broome R Corbett S Smith W. Emergency department visits, ambulance calls, and mortality associated with an exceptional heat wave in Sydney, Australia, 2011: a time-series analysis. Environ Health. 2012;11:3. 22273155 10.1186/1476-069X-11-3 PMC3292446

[8] Fujimoto M Nishiura H. Baseline scenarios of heat-related ambulance transportations under climate change in Tokyo, Japan. PeerJ. 2022;10:e13838. 35923895 10.7717/peerj.13838 PMC9341446

[9] Oka K Honda Y Phung VLH Hijioka Y. Prediction of climate change impacts on heatstroke cases in Japan’s 47 prefectures with the effect of long-term heat adaptation. Environ Res. 2023;232:116390. 37302741 10.1016/j.envres.2023.116390

[10] Sera F Hashizume M Honda Y. Air conditioning and heat-related mortality: a multi-country longitudinal study. Epidemiology. 2020;31:779–787. 33003149 10.1097/EDE.0000000000001241

[11] Davis LW Gertler PJ. Contribution of air conditioning adoption to future energy use under global warming. Proc Natl Acad Sci USA. 2015;112:5962–5967. 25918391 10.1073/pnas.1423558112 PMC4434761

[12] Anderson GB Bell ML. Lights out: impact of the August 2003 power outage on mortality in New York, NY. Epidemiology. 2012;23:189–193. 22252408 10.1097/EDE.0b013e318245c61c PMC3276729

[13] Lin S Fletcher BA Luo M Chinery R Hwang SA. Health impact in New York City during the Northeastern blackout of 2003. Public Health Rep. 2011;126:384–393. 21553667 10.1177/003335491112600312 PMC3072860

[14] Rajaram N Hohenadel K Gattoni L. Assessing health impacts of the December 2013 Ice storm in Ontario, Canada. BMC Public Health. 2016;16:544. 27401213 10.1186/s12889-016-3214-7 PMC4940759

[15] Greenwald PW Rutherford AF Green RA Giglio J. Emergency department visits for home medical device failure during the 2003 North America blackout. Acad Emerg Med. 2004;11:786–789. 15231473 10.1197/j.aem.2003.12.032

[16] Freese J Richmond NJ Silverman RA Braun J Kaufman BJ Clair J. Impact of a citywide blackout on an urban emergency medical services system. Prehosp Disaster Med. 2006;21:372–378. 17334182 10.1017/s1049023x00004064

[17] Kearns RD Wigal MS Fernandez A. The 2012 derecho: emergency medical services and hospital response. Prehosp Disaster Med. 2014;29:542–545. 25231139 10.1017/S1049023X14001034

[18] Geiger T Gütschow J Bresch DN Emanuel K Frieler K. Double benefit of limiting global warming for tropical cyclone exposure. Nat Clim Change. 2021;11:861–866.

[19] Casey JA Fukurai M Hernández D Balsari S Kiang MV. Power outages and community health: a narrative review. Curr Environ Health Rep. 2020;7:371–383. 33179170 10.1007/s40572-020-00295-0 PMC7749027

[20] Wang Z Hong T Li H. Informing the planning of rotating power outages in heat waves through data analytics of connected smart thermostats for residential buildings. Environ Res Lett. 2021;16:074003.

[21] Organization for Cross-regional Coordication of Transmission Operators J. Report on the quality of electricity supply in Japan. 2022. Available at: https://www.occto.or.jp/houkokusho/2021/files/denki_no_shitsu_2020_211117r.pdf.

[22] Potera C. Air conditioning use and heat-related deaths: how a natural disaster presented a unique research opportunity. Environ Health Perspect. 2017;125:104007. 29084392 10.1289/EHP2342 PMC5933305

[23] Guojun H Tanaka T. Energy saving may kill: evidence from the Fukushima nuclear accident. Am Econ J Appl Econ. 2023;15:377–414.

[24] Dominianni C Lane K Johnson S Ito K Matte T. health impacts of citywide and localized power outages in New York City. Environ Health Perspect. 2018;126:067003. 29894117 10.1289/EHP2154 PMC6084843

[25] Nakajima K Takane Y Fukuba S Yamaguchi K Kikegawa Y. Urban electricity–temperature relationships in the Tokyo metropolitan area. Energy Build. 2022;256:111729.

[26] Takane Y Nakajima K Yamaguchi K Kikegawa Y. Decarbonisation technologies can halve the nonlinear increase in electricity demand in densely populated areas due to climate change. Sustain Cities Soc. 2023;99:104966.

[27] Tokyo Electric Power Company Holdings. Power outage information (Japanese). Available at: https://teideninfo.tepco.co.jp. Accessed 23 October 2022.

[28] Silva JMCS Tenreyro S. The log of gravity. Rev Econ Stat. 2006;88:641–658.

[29] Santos Silva JMC Tenreyro S. Further simulation evidence on the performance of the Poisson pseudo-maximum likelihood estimator. Econ Letters. 2011;112:220–222.

[30] Wooldridge JM. Econometric Analysis of Cross Section and Panel Data. MIT Press; 2002;108(2):245–254.

[31] Seposo X Madaniyazi L Ng CFS Hashizume M Honda Y. COVID-19 pandemic modifies temperature and heat-related illness ambulance transport association in Japan: a nationwide observational study. Environ Health. 2021;20:122. 34857008 10.1186/s12940-021-00808-w PMC8637525

[32] Ng CFS Ueda K Takeuchi A. Sociogeographic variation in the effects of heat and cold on daily mortality in Japan. J Epidemiol. 2014;24:15–24. 24317342 10.2188/jea.JE20130051 PMC3872520

[33] Kim Y Gasparrini A Hashizume M Honda Y Ng CFS Armstrong B. Heat-Related mortality in japan after the 2011 fukushima disaster: an analysis of potential influence of reduced electricity consumption. Environ Health Perspect. 2017;125:077005. 28686555 10.1289/EHP493 PMC5744700

[34] Gasparrini A Guo Y Hashizume M. Changes in susceptibility to heat during the summer: a multicountry analysis. Am J Epidemiol. 2016;183:1027–1036. 27188948 10.1093/aje/kwv260 PMC4887574

[35] Armstrong B Sera F Vicedo-Cabrera AM. The role of humidity in associations of high temperature with mortality: a multicountry, multicity study. Environ Health Perspect. 2019;127:97007. 31553655 10.1289/EHP5430 PMC6792461

[36] Kim H Lee J-T Fong KC Bell ML. Alternative adjustment for seasonality and long-term time-trend in time-series analysis for long-term environmental exposures and disease counts. BMC Med Res Methodol. 2021;21:2. 33397295 10.1186/s12874-020-01199-1 PMC7780665

[37] Madaniyazi L Tobias A Kim Y Chung Y Armstrong B Hashizume M. Assessing seasonality and the role of its potential drivers in environmental epidemiology: a tutorial. Int J Epidemiol. 2022;51:1677–1686. 35639562 10.1093/ije/dyac115 PMC9557844

[38] Altman DG Bland JM. Interaction revisited: the difference between two estimates. BMJ. 2003;326:219. 12543843 10.1136/bmj.326.7382.219 PMC1125071

[39] Tanaka T Okamoto S. Increase in suicide following an initial decline during the COVID-19 pandemic in Japan. Nat Hum Behav. 2021;5:229–238. 33452498 10.1038/s41562-020-01042-z

[40] Lee W Ebi KL Kim Y. Heat-mortality risk and the population concentration of metropolitan areas in Japan: a nationwide time-series study. Int J Epidemiol. 2021;50:602–612. 33346831 10.1093/ije/dyaa245

[41] Tokyo Electric Power Holdings Co. L. Report of the Typhoon No. 15 Response Verification Committee; 2020

[42] Energy USDo. Emergency Situation Reports: Hurricane Sandy; 2012.

[43] Ji C Wei Y Mei H. Large-scale data analysis of power grid resilience across multiple US service regions. Nat Energy. 2016;1:16052.

[44] Feng K Ouyang M Lin N. Tropical cyclone-blackout-heatwave compound hazard resilience in a changing climate. Nat Commun. 2022;13:4421. 35907874 10.1038/s41467-022-32018-4 PMC9338923

[45] Matthews T Wilby RL Murphy C. An emerging tropical cyclone–deadly heat compound hazard. Nat Clim Change. 2019;9:602–606.

[46] Kovats RS Hajat S Wilkinson P. Contrasting patterns of mortality and hospital admissions during hot weather and heat waves in Greater London, UK. Occup Environ Med. 2004;61:893–898. 15477282 10.1136/oem.2003.012047 PMC1757853

[47] Schulte F Röösli M Ragettli MS. Heat-related cardiovascular morbidity and mortality in Switzerland: a clinical perspective. Swiss Med Wkly. 2021;151:w30013. 34519460 10.4414/SMW.2021.w30013

[48] Iñiguez C Royé D Tobías A. Contrasting patterns of temperature related mortality and hospitalization by cardiovascular and respiratory diseases in 52 Spanish cities. Environ Res. 2021;192:110191. 32980302 10.1016/j.envres.2020.110191

[49] Nitschke M Tucker GR Bi P. Morbidity and mortality during heatwaves in metropolitan Adelaide. Med J Aust. 2007;187:662–665. 18072911 10.5694/j.1326-5377.2007.tb01466.x

[50] Toosty NT Hagishima A Tanaka KI. Heat health risk assessment analysing heatstroke patients in Fukuoka City, Japan. PLoS One. 2021;16:e0253011. 34153053 10.1371/journal.pone.0253011 PMC8216561

[51] Gifford RM Todisco T Stacey M. Risk of heat illness in men and women: a systematic review and meta-analysis. Environ Res. 2019;171:24–35. 30641370 10.1016/j.envres.2018.10.020

[52] Achebak H Devolder D Ballester J. Trends in temperature-related age-specific and sex-specific mortality from cardiovascular diseases in Spain: a national time-series analysis. Lancet Planet Health. 2019;3:e297–e306. 31230996 10.1016/S2542-5196(19)30090-7

[53] Yang J Ou C-Q Ding Y Zhou Y-X Chen P-Y. Daily temperature and mortality: a study of distributed lag non-linear effect and effect modification in Guangzhou. Environ Health. 2012;11:63. 22974173 10.1186/1476-069X-11-63 PMC3511876

[54] Disaster Prevention and Crisis Management Department in Chiba prefecture. Typhoon No.15 in 2019 (report No.128). Available at: https://chiba.secure.force.com/services/apexrest/commonsfile/?fileid=00P0o00002IkaybEAB&key=vizcW4AjnXmnDs7VqtxVHas6kjJxEQnsP2TxrmyJ.

[55] Barron J. Lights go on after biggest blackout, but not without 2nd day of suffering. Available at: https://www.nytimes.com/2003/08/16/nyregion/blackout-overview-lights-go-after-biggest-blackout-but-not-without-2nd-day.html.

[56] Kennedy R. Thousands stranded on foot by crippled trains, crawling buses and traffic gridlock. Available at: https://www.nytimes.com/2003/08/15/us/blackout-2003-transportation-thousands-stranded-foot-crippled-trains-crawling.html.

[57] e-Stat. Regional Distribution of the Quantity and Diffusion Rate of Major Durable Consumer Goods per 1000 Households in the National Family Structure Survey. 2023. Available at: https://www.e-stat.go.jp/dbview?sid=0003108733.

