# Supplementary material for: Heat-related mortality and ambulance transport after a power outage in the Tokyo metropolitan area
Source: Environ Epidemiol. 2024 Feb 19;8(2):e292. doi: 10.1097/EE9.0000000000000292 (PMC11008645; doi:10.1097/EE9.0000000000000292)
Supplement: Supplementary file 1 [file ee9-8-e292-s001.pdf]

Supplementary materials

1. Data sources
    i. Climatic data
    ii. Outcome data
    iii. Electricity consumption and blackout cases
    iv. Blackout case
2. Prediction of typhoon-triggered electricity reduction
3. Sub-group analysis
4. Sensitivity analysis

1. *Data sources*

i. Outcome data

Daily cases of HIAT were gained at the fire department level disaggregated by sex, age groups (newborn, infant, children, adults, and elderly). To capture the characteristics of locations where HIAT cases are identified, we consider four categories: "home," "office or school," "public places," and "others." While "office or school" encompasses all HIAT cases in workplaces and schools, we have further subcategories within the office category, such as "workplace 1" for construction sites and factories, and "workplace 2" for fields, forests, and seas. "Public places" includes both indoor and outdoor public locations like restaurants, public transportation facilities, and parking lots. "Others" comprises cases that occurred on roads and in unknown places. Mortality data were obtained from the Ministry of Health, Labour and Welfare of Japan and we classified individual data into fire department-level data. We additionally set mortality into 20 subcategories according to ICD-10.

Subgroup characteristics

		Pre-power outage 7/1 - 9/8		Post-power outage 9/9 – 9/30	
		N	(%)	N	(%)
Heat-related illness ambulance transport					
Total		13479	-	1436	-
Place	Home	5427	(40.3)	515	(35.9)
	Office or school	2457	(18.2)	388	(27)
	Public places	2942	(21.8)	297	(20.7)
	Other*	2653	(19.7)	236	(16.4)
Sex	Male	8527	(63.3)	905	(63)
	Female	4952	(36.7)	530	(36.9)
Age	0-64	6594	(48.9)	745	(51.9)
	+65	6885	(51.1)	691	(48.1)
Mortality					
Total		56056	-	18008	-
Cause	Cardiovascular	13780	(24.6)	4245	(23.6)
	Respiratory	7492	(13.4)	2365	(13.1)
	Other	34784	(62.1)	11398	(63.3)
Sex	Male	30011	(53.5)	9498	(52.7)
	Female	26045	(46.5)	8510	(47.3)
Age	0-64	5893	(10.5)	1838	(10.2)

	+65	50163	(89.5)	16170	(89.8)

Table S1. Subgroup characteristics. Total number of each category in two periods – pre- and post- power outage.

*HIAT cases transported from places except for home, office, school, and public places inside and out. This category includes public places inside and outside (e.g., gyms, parks, shopping malls and sea) and roads.

ii. *Electricity consumption and power outage*

Hourly electricity consumption data at 1405 substations distributing power to central Japan provided by the TEPCO power grid. The spatial resolution was high, especially in the Tokyo Metropolitan Area[1]. For example, the average area covered by individual substations in the 23 wards of Tokyo was 1.86 km^2. In total, 115 substations were removed from the analysis because of low building densities (<50 buildings km^{-2}) or missing data. Thus, we used data from 1290 substations.

Electricity consumption Y_z in fire station area z (Fig. S2 color) is estimated using the substation's electricity consumption (Fig. S2 black line) as follows:

$$Y_z = \frac{\sum_{i=1}^{N} x_i \times F_{i,z}}{\sum_{i}^{N} F_{i,z}} Y_z = \sum_{i=1}^{N} x_i \times F_{i,z} \qquad (1)$$

where N is the number of substations, x_i is the electricity consumption per total floor area at substation i, and $F_{i,z}$ is the total floor area of i in z. The total floor area served by each substation was obtained from the building footprint and height (polygon) data of Esri Japan (https://www.esrij.com/products/data-content-geosuite-shosai/specifications/spec2020/)

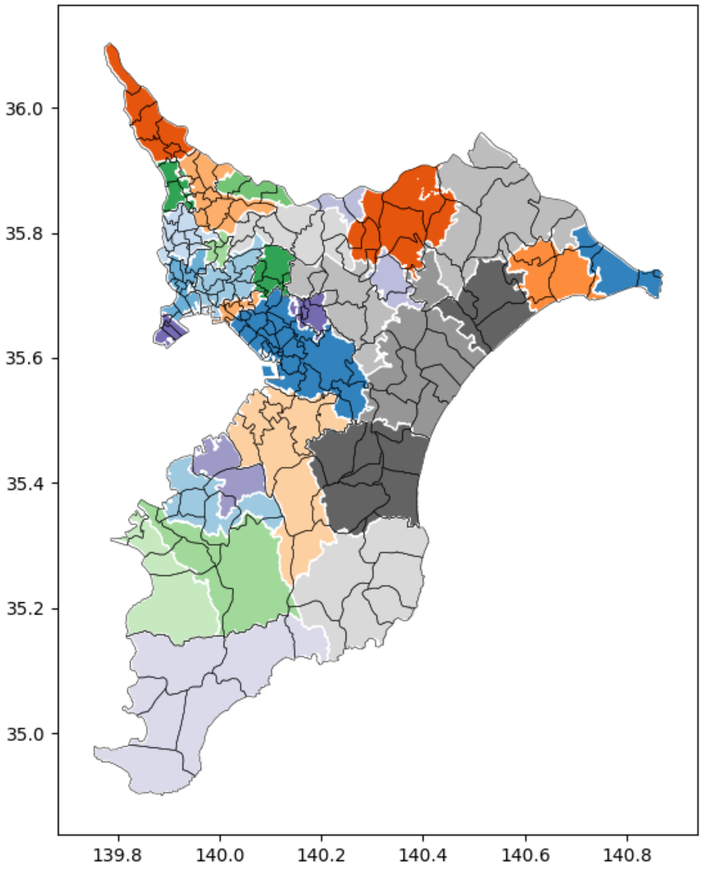

Figure S1. Areas of electricity substations (black line) and fire departments (white line with colored map) in the Kanto region of Japan.

iii. *Power outage*

Power outage at the city level was also collected from TEPCO. We assumed a power outage of more than 12 hours and aggregated the city-level data into fire department district-level data.

iv. *Meteorological data*

Our study area encompasses 7 prefectures in Kanto region of Japan. (Ibaraki, Tochigi, Gunma, Saitama, Chiba, Kanagawa, and Shizuoka) and 136 fire department districts are analysed. Daily meteorological data including daily mean air temperature and relative humidity were obtained from the Japan Meteorological Agency. We used daily meteorological data from the nearest weather station to each fire department. We excluded two weather stations (Okunikko and Fuji) for candidates because they were in high altitudes and people in the district are not supposed to live there. The trend of daily mean

temperature from 2015 to 2019 is shown (figure S1).

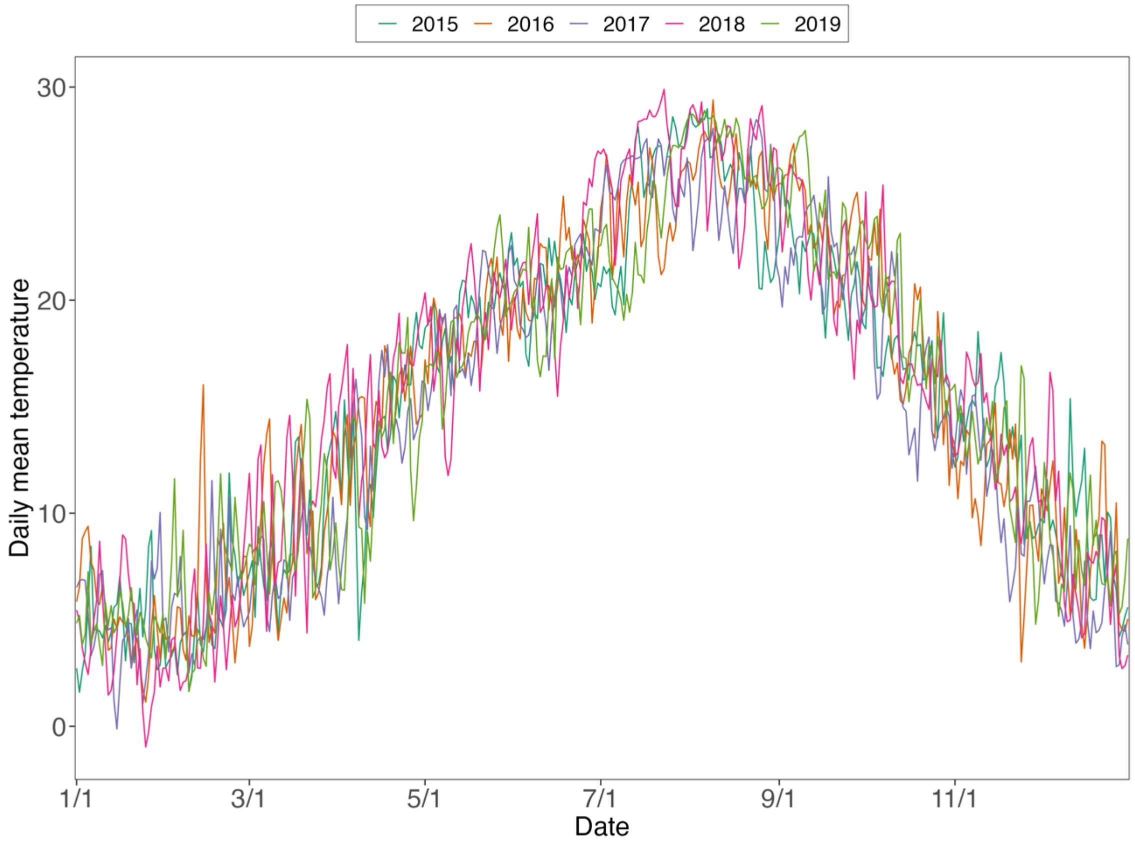

Figure S2. The trend of daily mean temperature from 2015 to 2019. The average daily mean temperature in the meteorological stations used in this study is plotted.

2. *Prediction for typhoon-triggered electricity reduction*

$$Y_t = \beta + \alpha_1 \times ns(\text{Temp}_t\ 3), +\alpha_2 \times \text{Humidity}_{\ t} + \alpha_3 \times \text{DOW}_t + \alpha_4 \times \text{Holiday}_t + \alpha_5 \times \text{Year}_t + \alpha_6 \times ns(\text{DOS}, 6) + \epsilon_t \quad (1)$$

Yt is the daily electricity consumption for each fire department. β is an intercept, Temp_t is daily mean temperature, and $\text{Humidity}_{\ t}$ is daily relative humidity. We adjusted day of week, holiday, and year as DOW_t, Holiday_t, and Year_t, respectively. A natural cubic spline with two equally spaced knots for temperature and five for the day of the year (seasonality) was used. ϵ_t is the normal-distributed error term. Each degree of freedom was decided based on the sum of RMSE for 136 fire departments in the validation period

– 2019 July to August. Fitted period is 2016 January to 2019 June.

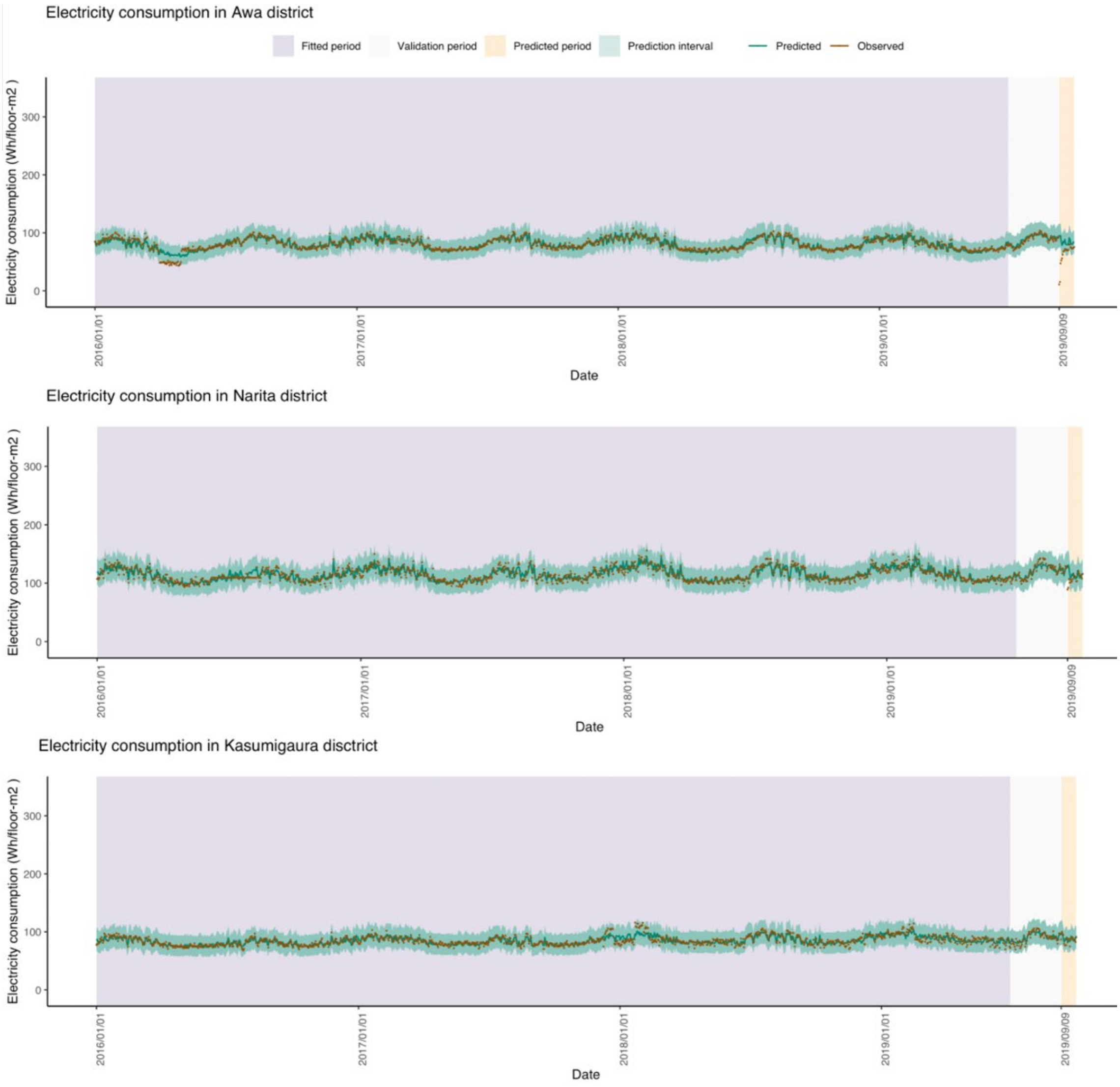

Figure S3. Predicted and observed electricity consumption.

3. *Sensitivity Analysis*

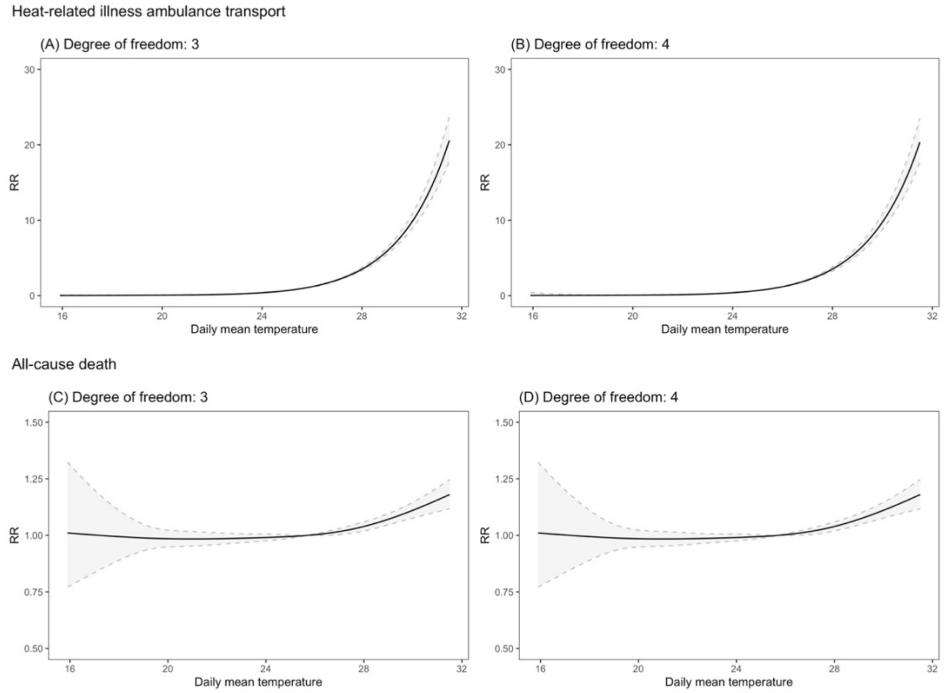

Figure S4. Sensitivity analysis for HIAT and all-cause mortality using the different degrees of freedom for the temperature variable in the baseline model at 0% electricity reduction. (A) The degree of freedom is 3 for HIAT (B) Degree of freedom is 4 for HIAT (C) Degree of freedom is 3 for all-cause mortality (D) Degree of freedom is 4 for all-cause mortality.

4. Sub-group analysis

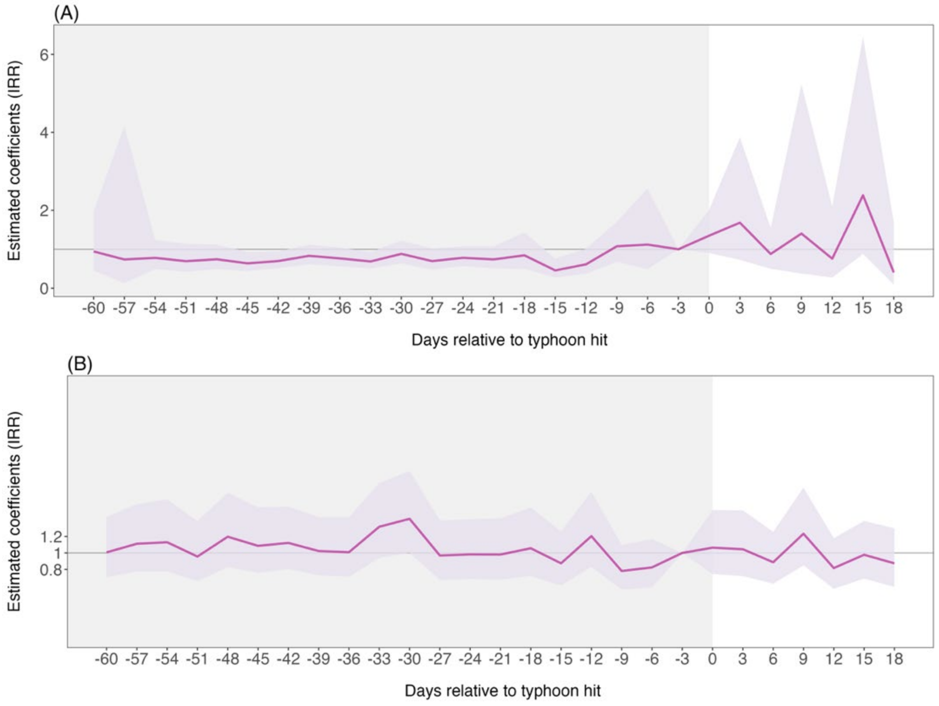

Figure S5. Subgroup analysis of event study for HIAT by age. (A) age 0-64 (B) age 65 and over

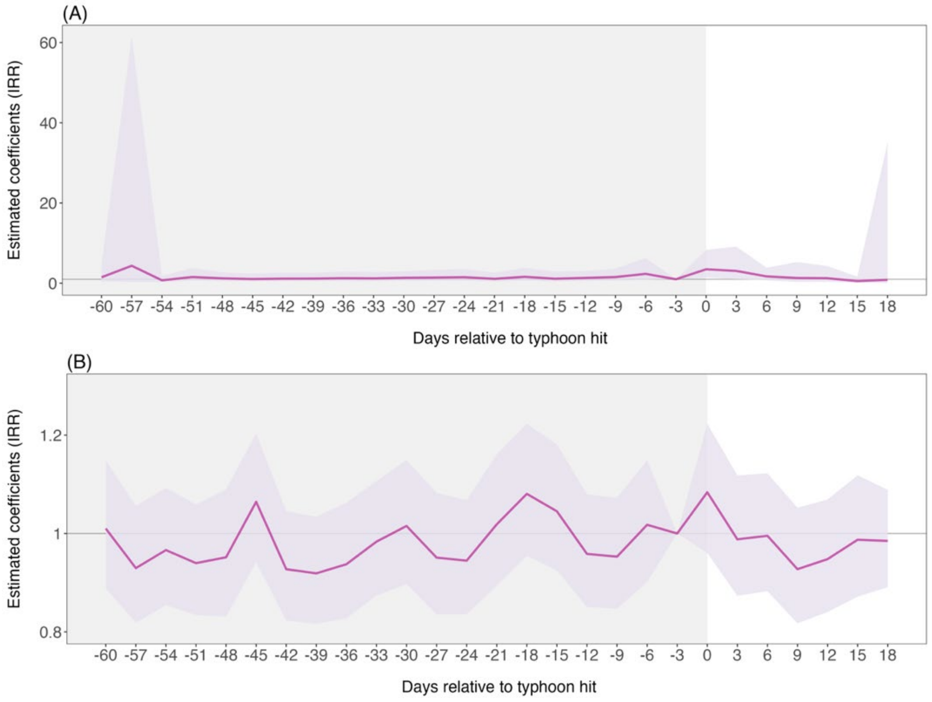

Figure S6. Subgroup analysis of event study for all-cause mortality by age (A) age 0-64

(B) age 65 and over

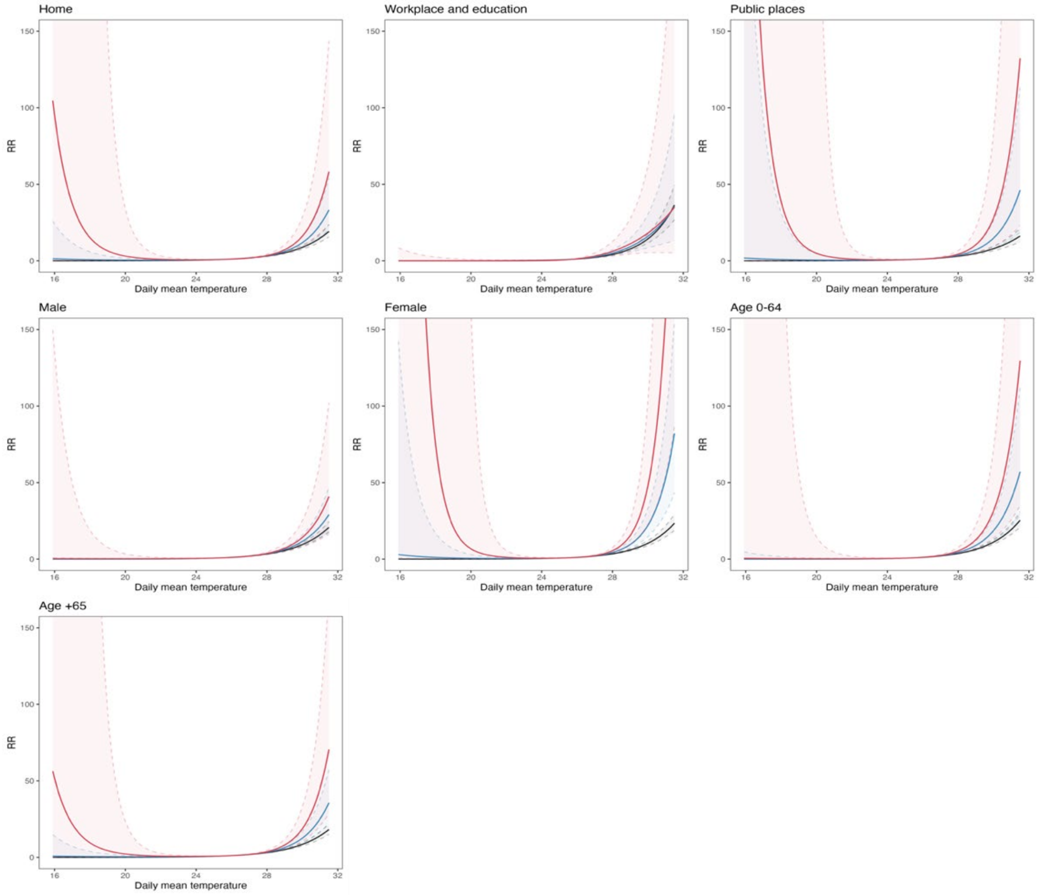

Figure S7. Subgroup analysis for HIAT by place, sex, and age

The black line shows 0% electricity reduction (ER), the blue line shows power outage at 10% ER, while the red line shows that at 20 % ER. The dotted lines represent 95% confidence intervals.

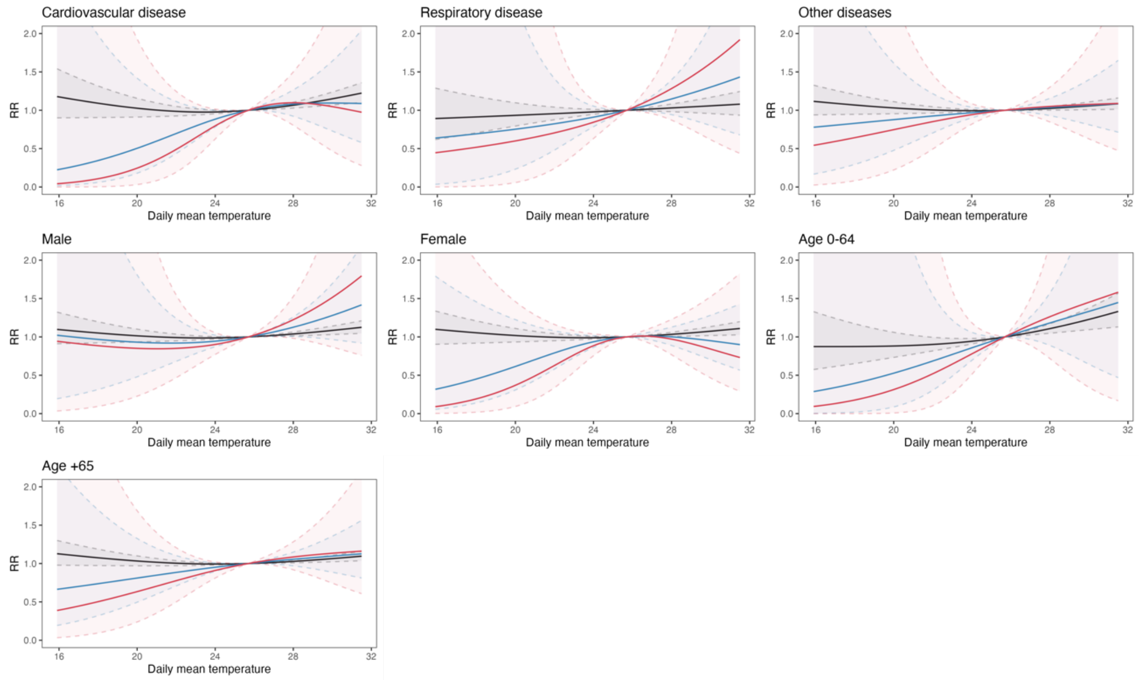

Figure S8. Subgroup analysis for death cases by disease, sex, and age

The black line shows 0% electricity reduction (ER), the blue line shows power outage at 10% ER, while the red line shows that at 20 % ER. The dotted lines represent 95% confidence intervals.

Reference

1. Nakajima K, Takane Y, Fukuba S, Yamaguchi K, Kikegawa Y. Urban electricity–temperature relationships in the Tokyo Metropolitan Area. *Energy and Buildings* 2022; **256**: 111729.
